# Transcriptional Profiling Reveals Differential Gene Expression of Amur Ide (*Leuciscus waleckii*) during Spawning Migration

**DOI:** 10.3390/ijms160613959

**Published:** 2015-06-18

**Authors:** Jun Cui, Jian Xu, Songhao Zhang, Kai Wang, Yanliang Jiang, Shahid Mahboob, Khalid A. Al-Ghanim, Peng Xu

**Affiliations:** 1 Chinese Academy of Fishery Sciences Key Laboratory of Aquatic Genomics, Centre for Applied Aquatic Genomics, Chinese Academy of Fishery Sciences, Beijing 100141, China; E-Mails: cuijun@mail.dlut.edu.cn (J.C.); xuj@cafs.ac.cn (J.X.); zsh891007@gmail.com (S.Z.); wangk@cafs.ac.cn (K.W.); jiangyl@cafs.ac.cn (Y.J.); 2 Beijing Key Laboratory of Fishery Biotechnology, Beijing 100141, China; 3 Department of Zoology, College of Science, King Saud University, P.O. Box 2455, Riyadh 11451, Saudi Arabia; E-Mails: mushahid@ksu.edu.sa (S.M.); kghanim@ksu.edu.sa (K.A.A.-G.); 4 Department of Zoology, GC University, Faisalabad 38030, Pakistan

**Keywords:** Amur ide (*Leuciscus waleckii*), comparative transcriptome, gene expression, freshwater, spawning migration

## Abstract

Amur ide (*Leuciscus waleckii*), an important aquaculture species, inhabits neutral freshwater but can tolerate high salinity or alkalinity. As an extreme example, the population in Dali Nor lake inhabits alkalized soda water permanently, and migrates from alkaline water to neutral freshwater to spawn. In this study, we performed comparative transcriptome profiling study on the livers of Amur ide to interrogate the expression differences between the population that permanently inhabit freshwater in Ganggeng Nor lake (FW) and the spawning population that recently migrated from alkaline water into freshwater (SM). A total of 637,234,880 reads were generated, resulting in 53,440 assembled contigs that were used as reference sequences. Comparisons of these transcriptome files revealed 444 unigenes with significant differential expression (*p*-value ≤ 0.01, fold-change ≥ 2), including 246 genes that were up-regulated in SM and 198 genes that were up-regulated in FW. The gene ontology (GO) enrichment analysis and KEGG pathway analysis indicated that the mTOR signaling pathway, Janus kinase-signal transducer and activator of transcription (JAK-STAT) signaling pathway, and oxidative phosphorylation were highly likely to affect physiological changes during spawning migration. Overall, this study demonstrates that transcriptome changes played a role in Amur ide spawning migration. These results provide a foundation for further analyses on the physiological and molecular mechanisms underlying Amur ide spawning migration.

## 1. Introduction

Amur ide (*Leuciscus waleckii*, Cyprinidae) live in the Heilongjiang River basin in Russia, Mongolia, China, and Korea. Amur ide inhabit freshwater but can tolerate high salinity and alkalinity [1]. For example, Amur ide that inhabits Dali Nor lake (43°22ʹ43ʹʹ N, 116°39ʹ24ʹʹ E) can survive in highly alkaline water up to a pH of 9.6 [2]. Spawning migration is a characteristic of Amur ide that live in Dali Nor lake. The Amur ide population in Dali Nor lake have developed special spawning migration behavior which migrates to freshwater in Ganneng Nor lake to spawn through the short Shali river in late April to early May [1].

Amur ide is economically important to local Mongolians who live near the Dali Nor lake and Ganggeng Nor lake [3]. Despite their economic importance, the physiological and molecular mechanisms underlying Amur ide spawning migration are still unknown. Very limited physiological and genetic studies have been performed, and very few genetic resources have been developed. So far, only a few genetic markers have been developed for population genetic evaluation and phylogenetic analysis [4,5,6]. The mitochondrial genome was completely sequenced and annotated, providing basic molecular tools for ecological and genetic studies [7]. Recently, high throughput transcriptome sequencing data were collected based on Illumina platform from both the freshwater population in Ganggeng Nor lake and the alkaline-tolerance population in Dali Nor lake. Expression profiles were compared between these two distinct populations, which discriminate a number of functional genes that associate with the hyperosmotic and alkaline adaptation. The preliminary study on this species provides us the genomic basis for further investigation of the mechanism of its alkaline tolerance as well as other physiological processes [1,2,8]. Amur ide has recently been developed as a potential aquaculture species in a wide distribution of fresh, saline, and alkaline waters in northern China. Additionally, Amur ide rapidly adapted to paleoenvironmental changes since the early Holocene; therefore, scientists are also interested in Amur ide microevolution mechanisms. Consequently, study of the physiological and molecular mechanisms of Amur ide spawning migration is important to optimize breeding and understand this species’ ability to adapt to environmental changes.

Next-generation sequencing-based RNA-Seq analysis is widely used for transcriptome expression profiling, gene identification, gene associated markers development, noncoding RNAs identification and profiling, alternative splicing analysis, *etc.*, [9,10,11,12,13]. In the past several years, RNA-Seq has been widely used for differential gene expression (DEG) analysis in a variety of teleosts. For example, RNA-Seq was used to unveil gene expression differences and pathways in response to various pathogenic infections in *Ictalurus punctatus* [14,15], under certain environmental stress in *I. punctatus* [11], tissue difference in *Takifugu rubripes* [16,17,18], sex difference in *I. punctatus* [19], ploidy differences in *Carassius auratus* [20], and skin color variation in *Cyprinus carpio* [21].

The liver was chosen for analysis because of its high metabolic activity and essential physiological roles in response to stress. In previous research, it was found that the liver played an important role in hyperosmotic and hypersaline conditions in the rainbow trout (*Oncorhynchus mykiss*) [22]. In addition, massive gene expression changes have been identified in the liver of many teleost species after stress challenges. For example, a total of 1099 DEGs with a cutoff of two-fold change were identified in the liver of catfish in response to heat stress [11]. Specifically, genes involved in oxygen transport, protein folding and degradation, and metabolic process were highly induced, whereas general protein synthesis was dramatically repressed in response to lethal temperature stress [11]. Thus, in the liver of fathead minnows, a total of 309 genes, which were differentially expressed, had more than two-fold change in expression level between control and municipal waste water effluent-exposed fish [23].

In this study, we used RNA-Seq to investigate genome-wide gene expression differences in the livers of Amur ide from different environments: one group that permanently inhabits the freshwater in Ganggeng Nor lake (FW), and the other is acute freshwater acclimation group which inhabits alkalized soda water in Dali Nor lake and recently migrated into the freshwater for spawning (SM). The DEGs were identified, annotated, and enriched. This study highlights those enrichment pathways (mammalian target of rapamycin (mTOR) signaling pathway, Janus kinase-signal transducer and activator of transcription (JAK-STAT) signaling pathway, and oxidative phosphorylation pathway) on Amur idebetween FW and SM. This study provides information to explain the underlying physiological and molecular mechanisms of Amur ide spawning migration.

## 2. Results

### 2.1. Sequencing of Short Expressed Reads

To better understand the physiological mechanisms underlying Amur ide spawning migration, we conducted a comparative transcriptome analysis between FW and SM using next-generation sequencing. The RNA samples from livers were collected and deep sequenced using Illumina HiSeq 2000. As shown in Table 1, a total of 637,234,880 paired-end reads were generated from two samples with 101 bp read lengths. Approximately 351,560,104 and 285,674,776 reads were generated for FW and SM, respectively. After removal of ambiguous nucleotides, low-quality sequences (Phred quality scores < 20), contaminated microbial sequences and ribosomal RNA sequences, a total of 466,917,416 cleaned reads (73.3%) were harvested for further analysis. There were 232,819,696 and 234,097,720 cleaned reads of FW and SM samples, respectively.

**Table 1:** Summary of the RNA-Seq data.

Group	Reads	Clean Reads	Mapped Reads	Mapping Ratio (%)
FW	351,560,104	232,819,696	194,404,446	83.5
SM	285,674,776	234,097,720	199,451,257	85.2
Total	637,234,880	466,917,416	393,855,703	84.4

### 2.2. Reference Assembly and Annotation

**Figure 1:**
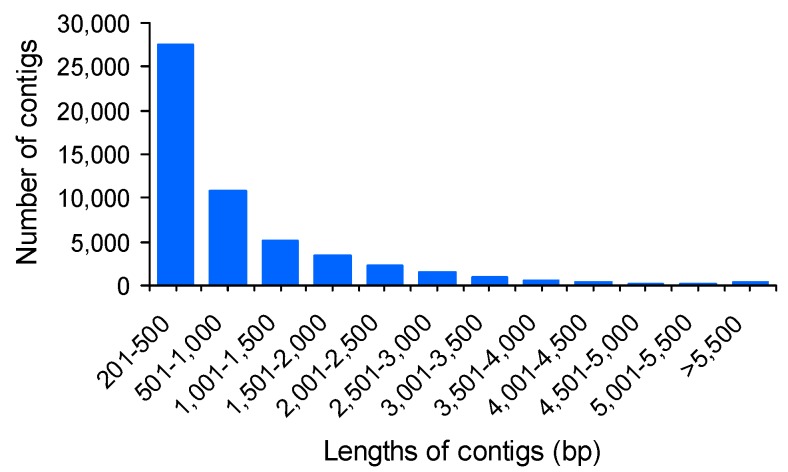
Distribution of assembled transcriptome contig lengths.

Numerous *de novo* assemblers have recently been developed to assemble RNA-Seq short reads [24]. In this study, all of the cleaned reads were pooled and *de novo* assembled to generate reference sequences using Trinity assembler [25]. After the removal of sequence redundancy using CD-HIT software, the 53,440 contigs, ranging from 201 to 13,690 bp in length, were generated as the reference sequences for subsequent analysis (Table 2). The average length was 917 bp, N50 length was 1686 bp, and median length was 476 bp. The contig length distribution is shown in Figure 1. To assess the quality of the sequencing and *de novo* assembly, all clean reads were mapped back to the assembled sequences. As shown in Table 1, the mapping ratios were 83.5% and 85.2% for FW and SM, respectively.

**Table 2:** Transcriptome reference assembly and annotation statistics.

Steps	Categories	Number/Length
**Assembly**	Number of contigs	53,440
	Maximum contig length	13,690 bp
	Minimum contig length	201 bp
	Average contig length	917 bp
	Median contig length	476 bp
	N50 length	1686 bp
**Annotation**	Contigs with blast hits to NR *	38,382
	Contigs with blast hits to UniProt	37,809
	Contigs with blast hits to Zebrafish proteins	38,559
	Unigenes predicted	19,972
	Unigenes with GO terms	10,988

* The “NR” is the abbreviation of non-redundant database.

Gene prediction was performed on the assembled contigs by BLASTx search against three protein databases, including the Ensembl zebrafish protein, UniProt-SwissProt (UniProt), and NCBI non-redundant (nr) protein databases using BLASTx with an *E*-value cutoff of 1 × 10^−10^. A total of 38,559 contigs had a significant BLAST hit against 16,949 unique zebrafish genes (unigenes). The number of matches to the UniProt database was 37,809 contigs with a putative 18,918 unigene matches. The number of matches to the nr database was 38,382 contigs with 18,526 putative unigenes. Cumulatively, a total of 19,972 unique genes had significant hits in at least one database (Table 2).

**Figure 2:**
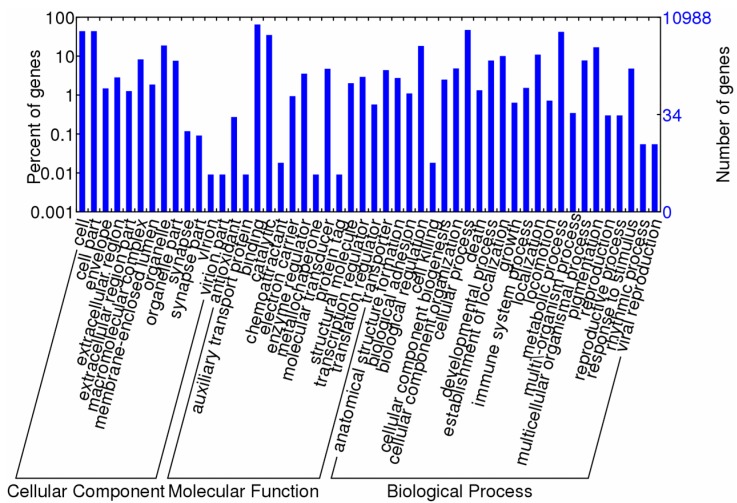
Gene ontology (level 2) for assembled transcriptome contigs under molecular functions, cellular components, and biological processes.

Gene ontology (GO) annotation was then performed using Blast2GO. A total of 10,988 unique proteins were assigned at least one GO term for describing biological processes, molecular functions, and cellular components (Figure 2). For biological processes, genes involved in the cellular process (47.1%) and metabolic process (42.1%) were highly represented. For molecular functions, binding (65.1%) was the most represented GO term, followed by catalytic activity (34.9%). The major categories of cellular components were cell part (43.8%) and cell (43.8%).

**Figure 3:**
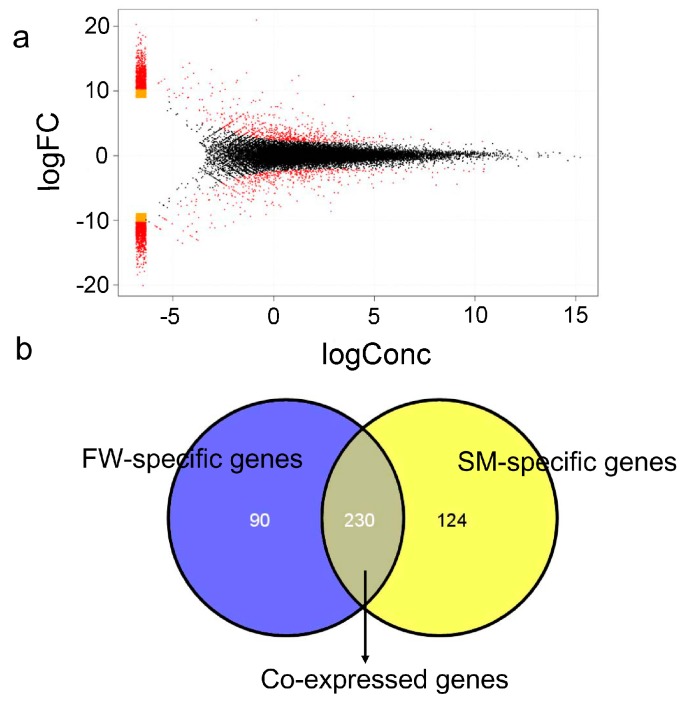
Differential expressed genes between fresh water (FW) and spawning migration (SM) livers. (**a**) MA (Log ratios–mean average) plots showing gene expression in the liver. The *y*-axis represents the logarithm of fold change and the *x*-axis represents the logarithm of transcript counts. M is the log ratio of the two dyes used in the hybridization, and A is the average of the log intensities; (**b**) Venn diagram displays the number of FW-specific, SM-specific, and co-expressed genes.

### 2.3. Differential Gene Expression (DEG) Identification

Based on the criteria that |fold-change| ≥ 2 and *p*-value ≤ 0.01, a total of 444 unigenes showed significant differential expression in the liver of the FW samples compared with the SM samples. Of these DEGs, 198 genes showed higher expression in livers of the FW samples and 246 genes showed higher expression in livers of the SM samples (Table S1). M-A plots were drafted using “eps” format files, as shown in Figure 3a. Venn diagram of the DEGs illustrated that the number of FW-specific, SM-specific, and co-expressed genes were 90, 124, and 230, respectively (Figure 3b).

**Figure 4:**
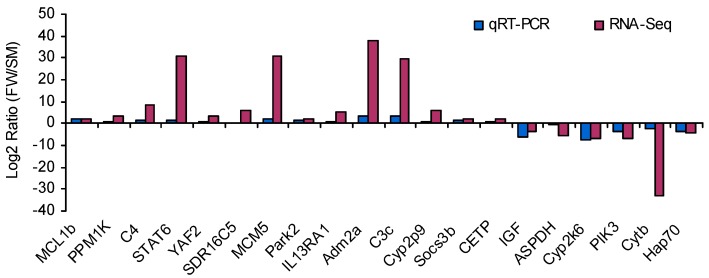
DEGs validated by qRT-PCR. Comparison of RNA-Seq and qRT-PCR validation results. *x*-axis shows genes in three tissues validated in this study; *y*-axis shows the log_2_ ratio of expression in FW (freshwater) *versus* SM (spawning migration) Amur ide. MCL1b, myeloid cell leukemia sequence 1b; PPM1K, protein phosphatase 1K; C4, complement component 4; STAT6, signal transducer and activator of transcription 6, interleukin-4 induced; YAF2,YY1 associated factor 2; SDR16C5, short chain dehydrogenase/reductase family 16C, member 5b; MCM5, minichromosome maintenance deficient 5; Park2, Parkin 2; IL13RA1, interleukin 13 receptor, α 1; Adm2a, adrenomedullin 2a; C3c, complement component c3c; Cyp2p9, cytochrome P450, family 2, subfamily P, polypeptide 9; Socs3b, suppressor of cytokine signaling 3b; CETP, cholesteryl ester transfer protein, plasma; IGF, Insulin-like growth factor; ASPDH, aspartate dehydrogenase domain containing; Cyp2k6, cytochrome P450, family 2, subfamily K, polypeptide 6; PIK3, phosphoinositide-3-kinase, regulatory subunit 1 (p85 α); Cytb, cytochrome b, mitochondrial; Hap70, heat shock cognate 70 kD protein, tandem duplicate 3.

To validate the DEGs identified by RNA-Seq expression analysis, we randomly selected 20 genes for quantitative real-time PCR (qRT-PCR) confirmation of differential expression. Fold changes from qRT-PCR were compared with RNA-Seq expression analysis results. As shown in Figure 4, the RNA-Seq results were confirmed by the qRT-PCR results, which support the reliability and accuracy of the RNA-Seq expression analysis.

### 2.4. Enrichment and Pathway Analysis

The 444 DEGs were categorized based on their likely function using Blast2GO. All of the DEGs were classified into biological processes, molecular functions, and cellular components. Together with the GO annotation of the reference sequences, the WEGO program (http://wego.genomics.org.cn) revealed eight GO enrichment terms at the second GO level (*p* < 0.05). These eight GO enrichment terms are shown in Table 3, and they included extracellular region (GO: 0005576), cell (GO: 0005623), macromolecular complex (GO: 0032991), organelle (GO: 0043226), organelle part (GO: 0044422), cell part (GO: 0044464), cellular component biogenesis (GO: 0044085), and binding (GO: 0005488). In the GO enrichment categories, 293 genes were considered most informative for further pathway analysis.

**Table 3:** The gene ontology (GO) enrichment results of genes with significantly different expression in SM and FW samples.

GO ID	GO Term	*p*-Value	Counts Reference/Differential Genes
GO: 0005576	Extracellular region	0.001	312/20
GO: 0005623	Cell	<0.001	4817/115
GO: 0032991	Macromolecular complex	0.007	907/14
GO: 0043226	Organelle	0.019	2062/46
GO: 0044422	Organelle part	0.031	836/15
GO: 0044464	Cell part	<0.001	4817/115
GO: 0044085	Cellular component biogenesis	0.026	274/2
GO: 0005488	Binding	0.001	7161/249

The “Counts” column indicates the number of genes associated with the GO term.

The pathway analysis was conducted using KEGG pathway analysis combined with literature searches. We mainly focused on the three pathways (*p* < 0.05) that underlie the physiological mechanism of Amur ide spawning migration. We included the (1) mTOR signaling pathway; (2) JAK-STAT signaling pathway; and (3) oxidative phosphorylation pathway. The key DEGs involved in each pathway are presented in Table 4.

**Table 4:** Detailed information regarding the DEGs involved in each of the three pathways.

Pathway Involved	Gene Name	Unigene ID	Fold Change
mTOR signaling pathway	*PIK3*	comp102544_c0	−6.51
	*RPS6Kba1*	comp102058_c22	−30.97
	*AKT2*	comp102966_c5	−29.68
	*IGF*	comp60482_c0	−3.79
	*DDIT4*	comp101409_c1	−31.58
JAK-STAT signaling pathway	*IL13RA1*	comp102860_c0	5.05
	*PIK3*	comp102544_c0	−6.51
	*IL7R*	comp101289_c0	−2.52
	*socs3b*	comp102087_c6	2.25
	*AKT2*	comp102966_c5	−29.68
	*STAT6*	comp102564_c2	30.61
Oxidative phosphorylation pathway	*COX1*	comp101302_c0	−32.02
	*Cytb*	comp98419_c0	−32.82
	*COX3*	comp99121_c0	−33.75
	*COX2*	comp125637_c0	−33.06
	*ATP6V1F*	comp89974_c0	−33.00
	*ATP6*	comp126569_c0	−32.62

## 3. Discussion

Many studies have reported mechanisms underlying physiological processes in fish such as heat stress response, energy metabolism, and the cell cycle [11]. However, knowledge regarding the physiological mechanisms underlying Amur ide spawning migration is limited. Recently, next-generation sequencing-based RNA-Seq analyses have dramatically changed the way functional complexity of transcriptomes in many organisms is investigated [26,27]. RNA-Seq is powerful for unraveling transcriptome complexity; identification of genes, gene associated markers, and regulatory non-coding RNAs; alternative splicing analysis; and transcriptome profiling [9,10,11]. To explore the physiological mechanisms underlying Amur ide spawning migration, livers were used to examine the expression profiles of the DEGs between FW and SM samples using high-throughput sequencing. In this study, functional enrichment analysis of differential genes was performed. Three pathways related to spawning migration were focused on, including the mTOR signaling pathway, JAK-STAT signaling pathway, and oxidative phosphorylation pathway. To the best of our knowledge, this is the first report of a comparative transcriptome analysis in spawning migration.

In mammal reproduction, female fertility is highly dependent on successful regulation of energy metabolism [28]. Moreover, the energy metabolism in rainbow trout changes during spawning; metabolism becomes increasingly aerobic, and the capacity for fatty acid utilization increases, which occurs concomitantly with phenotypic changes associated with sexual maturation [29]. During spawning, liver lipolysis was reduced and muscle glycogenolysis in the energy metabolism of the burbot (*Lota lota*) [30]. These results demonstrate the energy metabolism is strengthened during spawning migration. In this study, numerous genes, including *COX1-3*, *Cytb*, and *ATP6*, were more than 30-fold up-regulated in SM, indicating that oxidative phosphorylation has an active role in preparing energy for reproduction (Table 4). In summary, when Amur ide that inhabit soda lakes migrated to freshwater lakes and prepared to reproduce, their oxidative phosphorylation levels were enhanced, and a substantial amount of energy was expended.

Insulin-like growth factor (IGF) plays important roles during fish spawning migration, which can regulate osmotic pressure and consequently enhance fish adaptability. In previous studies, the plasma concentration of IGF-1 increased when *Salmo salar* was exposed to seawater [31]. In addition, transfer of smolting coho salmon to seawater was associated with increased gill IGF-1 mRNA [32]. This may be due to IGF-enhanced Na, K-activated ATPase expression [33,34]. IGF also plays a crucial role in reproduction. For example, IGF can inhibit the release of growth hormone (GH) and promote the production and release of gonadotropic hormone (GTH-II) [35]. In zebrafish, IGF may mediate the action of LH on oocyte maturation in zebrafish [36]. In this study, IGF expression was up-regulated in SM samples, indicating that IGF is involved in regulation of osmotic pressure and reproduction during the Amur ide spawning migration.

Previous studies revealed that changes in the osmolality of body fluids pose a serious danger to cells. For example, the apoptotic cells in the gills of tilapia significantly increased one day after transfer from freshwater to 70% seawater [37]. This result demonstrated that the change of osmotic pressure might activate some antiapoptosis signaling pathways to when Amur ide that inhabit soda lakes migrate to freshwater lakes to spawn. The phosphoinositide 3-kinase AKT (PI3K–AKT) network, which is activated by the mTOR or JAK-STAT signaling pathways, mediates intracellular signals to regulate a variety of cellular responses, including antiapoptosis, proliferation, and cell cycling [38]. Our results revealed that the PI3K and AKT2 genes involved in the PI3K–AKT network had up-regulated expression in SM ((Figure S1), which indicates that PI3K–AKT is a strong candidate for apoptosis regulation during Amur ide spawning migration. IGF in the mTOR signaling pathway, and interleukin 7 receptor (IL7R) in the JAK-STAT signaling pathway may activate the PI3K–AKT network [39,40,41]. Our results also revealed that IGF and IL7R were 3.79- and 2.52-fold up-regulated in SM, respectively, indicating that IGF and IL7R may be involved in the physiological mechanisms underlying Amur ide spawning migration and activate the PI3K–AKT network.

## 4. Materials and Methods

### 4.1. Ethics Statement

This study was approved by the Animal Care and Use committee of Center for Applied Aquatic Genomics at Chinese Academy of Fishery Sciences (Beijing, China). All surgery was performed under sodium pentobarbital anesthesia, and all efforts were made to minimize suffering.

### 4.2. Sample Collection

A total of 10 individuals (five males and five females) undergoing spawning migration (SM) were sampled in a freshwater stream of the Shali River (43°22ʹ43ʹʹ N, 116°39ʹ24ʹʹ E) after they migrated from the soda lake Dali Nor (43°22ʹ43ʹʹ N, 116°39ʹ24ʹʹ E) to the freshwater lake Ganggeng Nor. Another 10 individuals (five males and five females) that permanently inhabit freshwater were sampled from the Ganggeng Nor (FW). Individuals of both groups weighed 130–150 grams. Livers were dissected and collected. Tissue samples were stored in RNAlater (Qiagen, Hilden, Germany) and transported to the laboratory in Beijing at room temperature, then stored at −20 °C prior to RNA extraction.

### 4.3. RNA Extraction, Library Construction, and Sequencing

Total RNA was extracted from livers using Trizol Kit (Invitrogen, Carlsbad, CA, USA) according to the manufacturer’s instructions. RNA samples were treated with DNase I to remove genomic DNA. RNA concentration and integrity were checked using a Bioanalyzer 2100 with RNA 6000 Nano Labchips (Agilent Technologies, Santa Clara, CA, USA). Equal amounts of high quality RNA samples from the liver were then pooled for RNA-Seq analysis.

RNA-Seq library preparation and sequencing were carried out by HudsonAlpha Genomic Services Lab (Huntsville, AL, USA). cDNA libraries were prepared with 2.5 µg of starting total RNA following the protocols of the Illumina TruSeq RNA Sample Preparation Kit (Illumina, San Diego, CA, USA). The final library had an average fragment size of 270 bp and final yield of 400 ng. After KAPA quantitation and dilution, the library was sequenced on an Illumina HiSeq 2000 with 101 bp paired-end reads.

### 4.4. Sequence Data Processing and De Novo Assembly

Adaptor sequences were trimmed, and low quality reads and read length less than 10 bp were removed using CLC genomics Workbench (CLC Bio, Aarhus, Denmark). Trinity was used to assemble all cleaned reads with default parameters [25] and generate reference sequences for comparative transcriptome study.

### 4.5. Transcriptome Annotation and Ontology

The assembled reference transcriptome contigs were used as query sequences to search against the NCBI nr protein, UniProt-SwissProt (UniProt), and Ensembl zebrafish protein databases using BLASTx with an *E*-value cutoff of 1 × 10^−10^. GO annotation analysis was performed using Blast2GO software, which is an automated tool for the assignment of GO terms [42]. The annotation result was categorized with respect to biological process, molecular function, and cellular component at level 2.

### 4.6. DEG Analysis

All cleaned reads were mapped to the assembled reference transcriptome by Bowtie [43]. RSEM (RNA-Seq expression estimation by Expectation-Maximization) was used to estimate and quantify the gene and isoform abundances according to the Trinity-assembled transcriptome. Finally, we used edgeR to normalize the expression levels in each sample and obtain the differentially expressed transcripts by pairwise comparisons [44]. Transcripts with fold change >2 and *p* < than 0.01 were included in subsequent analyses as the DEGs.

### 4.7. Experimental Validation by qRT-PCR

qRT-PCR was used to validate the RNA-Seq results on 20 randomly selected gene accessions. The *β-actin* gene was used as an internal reference, and primers were designed using Primer5 software (Table S2). RNA samples from FW and SM (with three replicates per group) were used for qRT-PCR. First-strand cDNA was synthesized by SuperScript™ III First-strand Synthesis System for RT-PCR (Invitrogen, Carlsbad, CA, USA) following the manufacturer’s protocols. Briefly, qRT-PCR was performed in the optical 96-well plates with an ABI PRISM 7500 Real-time Detection System (Life Technology, Grand Island, NY, USA). The amplification was performed in a total volume of 15 μL, containing 7.5 μL 2× SYBR Green Master Mix reagent (Life Technology), 1 μL of cDNA (100 ng/μL), and 0.3 μL of 10 μM of each gene-specific primer. PCR was conducted as follows: 50 °C for 2 min, 95 °C for 10 min, 45 cycles of 95 °C for 15 s, and 60 °C for 1 min. After PCR, data were analyzed with ABI 7500 SDS software. The comparative *C*t method (2^−∆∆*C*t^ method) was used to analyze target gene expression. All data are shown at levels relative to the expression of the *β-actin* gene.

### 4.8. GO Enrichment Analysis

We performed enrichment analysis of significantly expressed GO terms using the Pearson χ-square test in the web-based program WEGO [45]. The differences of the frequency of assignment of GO terms in the DEG sets were compared with the overall transcriptome assembly. The threshold was set to *p* < 0.05. KEGG pathway analysis was also performed using DAVID (*p* < 0.05) based on GO enrichment analysis.

## 5. Conclusions

We performed comparative transcriptome profiling study of the livers of Amur ide from different environments: one group that permanently inhabits the freshwater of Ganggeng Nor lake, and the other that recently migrated to freshwater for spawning. A relatively large number of genes that displayed distinct expression differences in the liver were identified. The mTOR signaling pathway, JAK-STAT signaling pathway, and oxidative phosphorylation pathway were highly likely to affect physiological changes during spawning migration. These results provide a foundation for further analyses on the physiological and molecular mechanisms underlying Amur ide spawning migration.

## Supplementary Materials

Supplementary materials can be found at http://www.mdpi.com/1422-0067/16/06/13959/s1.
