# Supplementary material for: Transcriptional Profiling Reveals Differential Gene Expression of Amur Ide (Leuciscus waleckii) during Spawning Migration
Source: Int J Mol Sci. 2015 Jun 18;16(6):13959–72. doi: 10.3390/ijms160613959 (PMC4490533; doi:10.3390/ijms160613959)
Supplement: Supplementary file 1 [file ijms-16-13959-s001.pdf]

Supplementary Information

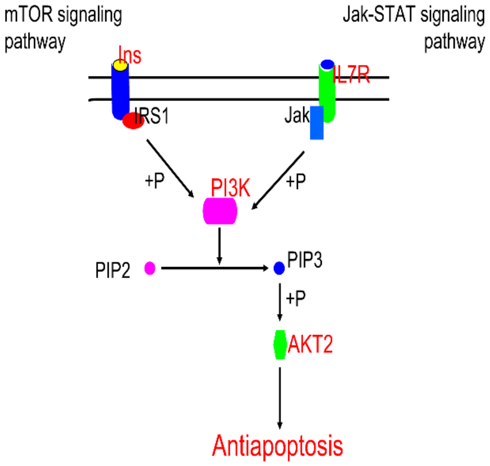

Figure S1. Diagram of putative PI3K–AKT network during Amur ide spawning migration. The *PI3K* and *AKT2* genes involved in the PI3K–AKT network and their expressions were up-regulated in SM, indicating that the PI3K–AKT is a strong candidate for apoptosis regulation and plays the critical roles on inhibition of apoptosis because of change of osmotic pressure during Amur ide spawning migration.

Table S1. Differential expressed genes identified by transcriptome comparison.

Gene ID	RSEM (FW)	RSEM (SM)	Fold Change	*p*-Value	Gene Name
comp99124_c0	56.67	0	37.78900814	3.65E-26	adrenomedullin 2a precursor
comp102945_c4	19.5	0	37.10132625	4.23E-24	PREDICTED: hypothetical protein LOC100333395
comp102927_c7	12.6	0	35.03081174	6.28E-18	Cideb protein
comp102325_c0	15.21	0	34.77221764	3.60E-17	MHC class II antigen
comp102117_c5	16.92	0	34.5338489	1.80E-16	starmaker
comp102025_c11	4.33	0	34.02053679	5.72E-15	ATP-dependent RNA helicase DDX54
comp102514_c3	3.68	0	33.96960035	7.93E-15	ubiquitin carboxyl-terminal hydrolase 16
comp102751_c15	9.11	0	33.79345644	2.61E-14	PREDICTED: hypothetical protein LOC566559 isoform 1
comp102456_c1	13.07	0	33.5835665	1.05E-13	PREDICTED: urokinase plasminogen activator surface receptor-like
comp102333_c8	7.1	0	33.41925976	3.08E-13	PREDICTED: GTPase IMAP family member 2-like
comp99484_c0	10.41	0	33.3858929	3.85E-13	PREDICTED: mpv17-like protein-like
comp100385_c0	3.87	0	33.28605528	7.18E-13	Thymidine phosphorylase
comp102998_c8	1	0	33.09450008	2.47E-12	PREDICTED: hypothetical protein LOC100537129
comp99539_c0	4.1	0	33.04307155	3.57E-12	glia maturation factor beta
comp102831_c4	15	0	32.71952708	2.72E-11	complement C3-H1
comp102845_c1	9.86	0	32.64407485	4.36E-11	uncharacterized protein LOC100006475 precursor
comp102924_c1	3.75	0	32.60690333	5.56E-11	PREDICTED: uncharacterized protein LOC100889275
comp102762_c2	1.39	0	32.34317329	2.99E-10	influenza virus NS1A-binding protein homolog B
comp100080_c1	1.27	0	32.1678557	8.83E-10	Zinc finger CCCH-type containing 14
comp102296_c3	4.36	0	32.13915184	1.04E-09	Uncharacterized protein
comp102351_c2	0.94	0	32.1288099	1.13E-09	PREDICTED: hypothetical protein LOC100537969
comp101989_c4	2.94	0	32.12229096	1.13E-09	BET1 homolog
comp101544_c1	3.86	0	31.94454258	3.26E-09	CD2 family receptor
comp102812_c4	6.25	0	31.8665226	5.26E-09	Uncharacterized protein

Table S1. *Cont.*

Gene ID	RSEM (FW)	RSEM (SM)	Fold Change	*p*-Value	Gene Name
comp100941_c2	1.68	0	31.8415772	5.80E-09	PREDICTED: magnesium transporter MRS2 homolog, mitochondrial-like
comp100775_c0	1.84	0	31.81292361	7.08E-09	Family with sequence similarity 82, member A2
comp102365_c3	4.67	0	31.78245833	8.67E-09	polyprotein
comp102497_c3	5.09	0	31.77558036	8.67E-09	PREDICTED: hypothetical protein LOC100007935
comp101653_c0	1.22	0	31.6826713	1.47E-08	complement C1q tumor necrosis factor-related protein 1 precursor
comp102571_c24	1.31	0	31.67795012	1.64E-08	PREDICTED: protein FAM20A-like, partial
comp100076_c7	2.35	0	31.6010296	2.56E-08	Ighmbp2l protein
comp102770_c0	0.84	0	31.51893121	4.09E-08	PREDICTED: hypothetical protein LOC100538314
comp100952_c3	3.64	0	31.49099552	4.62E-08	PREDICTED: desmoglein-4-like
comp102578_c7	3.22	0	31.47082977	5.22E-08	PREDICTED: complement C3
comp102753_c2	0.43	0	31.45126377	5.90E-08	Ugdh protein
comp101848_c2	0.71	0	31.31437987	1.28E-07	PREDICTED: transmembrane protein 56-B
comp102866_c10	0.87	0	31.23303391	1.94E-07	S-adenosylmethionine synthetase isoform type-2
comp102134_c8	1.6	0	30.99688645	7.45E-07	pyrroline-5-carboxylate reductase
comp101696_c1	1.35	0	30.81859709	1.72E-06	ribonuclease P protein subunit p14
comp102333_c9	3.61	0	30.81722375	1.72E-06	uncharacterized protein LOC406638
comp102898_c4	0.56	0	30.80133564	2.05E-06	DNA replication licensing factor MCM5
comp100708_c0	1.41	0	30.77540357	2.05E-06	dnaJ homolog subfamily C member 15
comp101267_c0	0.98	0	30.66063339	4.28E-06	thymidine kinase 2, mitochondrial
comp101889_c0	1.02	0	30.61828384	5.19E-06	AN1-type zinc finger protein 2B
comp102564_c2	0.5	0	30.60918662	5.19E-06	signal transducer and activator of transcription 6, interleukin-4 induced
comp88163_c0	3.08	0	30.59483133	5.19E-06	PREDICTED: hypothetical protein LOC100704550
comp102369_c3	1.72	0	30.56650261	6.32E-06	PREDICTED: TGF-beta receptor type-2-like
comp102539_c4	2.63	0	30.47061139	9.48E-06	PREDICTED: hypothetical protein LOC560673
comp102365_c2	2.54	0	30.37910264	1.45E-05	protease/polymerase
comp102110_c2	0.3	0	30.32069364	2.25E-05	pl10
comp95792_c0	2.67	0	30.28288732	2.25E-05	PREDICTED: hypothetical protein LOC100537242
comp102093_c0	0.52	0	30.25272535	2.82E-05	uncharacterized protein LOC368688
comp102630_c4	1.69	0	30.24559589	2.82E-05	PREDICTED: hypothetical protein LOC100334801
comp101797_c2	1.77	0	30.23226125	2.82E-05	PREDICTED: hypothetical protein LOC569383
comp102615_c8	0.39	0	30.17014366	4.53E-05	midnolin
comp68714_c0	3.61	0	30.12534605	4.53E-05	polymerase
comp95550_c0	2.66	0	30.12534605	4.53E-05	predicted protein
comp102528_c7	0.4	0	30.09052649	5.79E-05	PREDICTED: collagen alpha-1(XIII) chain
comp93184_c0	2.26	0	30.00986883	7.45E-05	PREDICTED: serine incorporator 3-like
comp93876_c0	2.9	0	29.94846828	9.64E-05	PREDICTED: hypothetical protein LOC100493142
comp102994_c7	0.97	0	29.91932194	1.26E-04	PREDICTED: protein NLRC3-like
comp102214_c4	2.16	0	29.89022784	1.26E-04	PREDICTED: alpha-2-macroglobulin
comp93215_c1	2.38	0	29.88433795	1.26E-04	pol-like protein ENS-3
comp101788_c18	0.25	0	29.8457929	1.65E-04	glycoprotein A repetitions predominant precursor
comp100654_c2	1.1	0	29.82749209	1.65E-04	TPA_exp: putative tyrosine recombinase
comp102365_c1	2.27	0	29.67283384	2.91E-04	gag-pol polyprotein
comp98009_c3	2.48	0	29.67283384	2.91E-04	PREDICTED: hypothetical protein LOC100695311
comp102646_c3	0.19	0	29.67207433	2.91E-04	Lipin 1
comp102073_c1	0.14	0	29.63045524	3.91E-04	Si:dkey-102f14.5

Table S1. *Cont.*

Gene ID	RSEM (FW)	RSEM (SM)	Fold Change	*p*-Value	Gene Name
comp102572_c6	3.36	0	29.59483133	3.91E-04	complement C3-S
comp99185_c2	2.55	0	29.59483133	3.91E-04	PREDICTED: hypothetical protein LOC100120441
comp90720_c0	1.78	0	29.42490633	7.27E-04	PREDICTED: tumor necrosis factor receptor superfamily member 11B
comp102296_c4	2.38	0	29.33179692	1.01E-03	PREDICTED: immunoglobulin superfamily member 3-like
comp102842_c9	1.91	0	29.33179692	1.01E-03	PREDICTED: retrotransposable element Tf2 155 kDa protein type 1-like
comp102826_c0	1.16	0	29.20206299	2.00E-03	uncharacterized protein LOC550566
comp100349_c1	2.56	0	29.12534605	2.00E-03	Uncharacterized protein
comp102333_c6	1.93	0	29.12534605	2.00E-03	PREDICTED: GTPase IMAP family member 8-like
comp68714_c1	2.01	0	29.12534605	2.00E-03	gag-pol polyprotein
comp91271_c0	1.76	0	29.12534605	2.00E-03	PREDICTED: RETRotransposon-like family member (retr-1)-like
comp102672_c4	0.13	0	29.0594996	2.86E-03	unnamed protein product
comp101324_c1	0.12	0	29.04196967	2.86E-03	serine/threonine-protein kinase SIK1
comp87594_c1	1.57	0	29.00986883	2.86E-03	PREDICTED: serine/threonine-protein kinase TAO3-like isoform 1
comp91133_c0	1.91	0	29.00986883	2.86E-03	PREDICTED: uncharacterized protein K02A2.6-like
comp99329_c2	1.85	0	29.00986883	2.86E-03	PREDICTED: hypothetical protein LOC100536880
comp102761_c1	2.11	0	14.27128118	3.75E-12	protein LSM14 homolog A
comp102185_c6	3.7	0	11.20599063	1.89E-12	PREDICTED: SLIT-ROBO Rho GTPase-activating protein 3
comp102031_c0	152.36	0.27	9.134886633	1.03E-21	ornithine decarboxylase 1a
comp102721_c6	0.3	0	8.663682495	2.45E-06	PREDICTED: copper-transporting ATPase 2
comp102791_c0	1.54	0	8.50670801	5.57E-12	complement C4-1
comp102875_c2	19.83	0.08	7.870392595	3.41E-17	PREDICTED: HHIP-like 1
comp67791_c0	13.08	0.07	7.571655518	4.65E-13	PREDICTED: SAM domain and HD domain-containing protein 1-like
comp102128_c0	0.65	0	7.525902929	1.45E-05	tryptophanyl-tRNA synthetase, cytoplasmic
comp103038_c1	5.18	0.03	7.514501174	2.40E-12	complement factor H-like 1 protein
comp102343_c2	5.63	0.06	6.589244915	4.22E-13	novel transglutaminase family protein
comp102329_c0	0.66	0.01	6.546120426	6.32E-06	PREDICTED: hypothetical protein LOC100701766
comp102608_c5	1.37	0.02	6.40138073	7.11E-08	PREDICTED: very long-chain acyl-CoA synthetase isoform 1
comp103011_c3	10.38	0.14	6.230045058	3.30E-08	Si:dkey-19a16.11
comp102949_c3	0.98	0.02	5.963676365	5.30E-08	PREDICTED: hypothetical protein LOC100008302
comp102612_c3	1.91	0.03	5.916709147	7.19E-10	PREDICTED: solute carrier organic anion transporter family member 1C1-like
comp101330_c3	17.22	0.33	5.691247748	2.33E-11	short chain dehydrogenase/reductase family 16C, member 5b
comp102986_c4	0.59	0.01	5.647870838	8.44E-05	cytochrome P450, family 2, subfamily P, polypeptide 9
comp102860_c0	1.38	0.04	5.049164533	5.82E-07	interleukin 13 receptor, alpha 1 precursor
comp102750_c4	33.55	1.03	5.026203033	3.25E-10	PREDICTED: hypothetical protein LOC100334998
comp54337_c0	2.53	0.1	4.605871234	4.88E-04	hypothetical protein BRAFLDRAFT_201218
comp93506_c1	2.13	0.1	4.342836828	1.33E-03	PREDICTED: retrovirus polyprotein, putative-like
comp102203_c0	4.56	0.23	4.300823326	2.79E-06	PREDICTED: hypothetical protein LOC100690401
comp102753_c9	0.48	0.03	4.165792527	1.33E-03	PREDICTED: nuclear factor 1 X-type

Table S1. *Cont.*

Gene ID	RSEM (FW)	RSEM (SM)	Fold Change	*p*-Value	Gene Name
comp102036_c0	2.47	0.14	4.108371574	3.04E-03	PREDICTED: hypothetical protein LOC100710874
comp95589_c1	2.49	0.14	4.108371574	3.04E-03	PREDICTED: ADM2
comp96263_c2	2.16	0.13	4.108371574	3.04E-03	PREDICTED: zinc finger protein 40
comp103139_c0	1137.15	68.06	4.062422971	3.93E-08	prostaglandin D2 synthase, brain precursor
comp102388_c8	7.08	0.43	4.051935629	2.46E-07	ankyrin repeat and protein kinase domain-containing protein 1
comp102817_c2	3.96	0.24	4.050835594	1.22E-05	aig2-like domain-containing protein 1-b precursor
comp59020_c0	4.21	0.25	4.050656076	6.41E-05	preprogalanin 2A
comp99861_c1	5.16	0.31	4.043556638	5.42E-07	pleckstrin homology domain containing, family G (with RhoGef domain) member 5a (plekhg5a), transcript variant X1
comp98319_c0	20.39	1.3	3.968152679	3.31E-07	novel protein similar to vertebrate mitochondrial ribosomal protein S14
comp102181_c0	2.6	0.17	3.944240476	6.51E-06	Palmdelphin b
comp102842_c5	2.23	0.16	3.828263655	7.47E-04	PREDICTED: retrotransposable element Tf2 155 kDa protein type 1-like
comp102643_c6	4.03	0.29	3.796561338	1.40E-05	reverse transcriptase
comp97269_c0	2.52	0.18	3.775796235	9.21E-04	Neuropilin 2b
comp102952_c8	2.78	0.22	3.664764923	1.42E-03	PREDICTED: dnaJ homolog subfamily C member 16
comp62047_c1	10.03	0.82	3.619677033	2.59E-06	IFN-inducible and antiviral protein
comp102842_c2	4.2	0.34	3.605871234	3.80E-05	PREDICTED: retrotransposable element Tf2 155 kDa protein type 1-like
comp102434_c7	0.89	0.07	3.600214508	8.98E-06	testis-expressed sequence 2 protein
comp102527_c0	1.2	0.1	3.585144068	6.51E-05	growth hormone receptor b
comp100295_c0	2.69	0.23	3.565229249	5.72E-04	unnamed protein product
comp101835_c0	1	0.09	3.552639785	1.10E-04	PREDICTED: toll-like receptor 5-like
comp93418_c0	3.28	0.28	3.539396284	1.96E-05	YY1-associated factor 2
comp102476_c2	2.55	0.22	3.521710786	6.82E-04	PREDICTED: transmembrane protein 80-like
comp102279_c14	6.26	0.55	3.496879853	3.51E-06	PREDICTED: protein phosphatase 1K, mitochondrial-like
comp94235_c1	1.85	0.17	3.480340352	2.81E-03	PREDICTED: apolipoprotein B-100
comp102262_c14	94.14	8.6	3.452010406	1.73E-06	apoptosis-inducing factor, mitochondrion-associated 2
comp102728_c3	7.29	0.67	3.445930321	6.77E-06	PREDICTED: hypothetical protein LOC100004944
comp102455_c10	4.38	0.42	3.394367128	1.16E-05	PREDICTED: plectin-like
comp92134_c0	2.7	0.27	3.342836828	1.42E-03	uncharacterized protein LOC768162
comp70094_c0	5.78	0.58	3.316841619	1.33E-05	PREDICTED: UMP-CMP kinase 2, mitochondrial, partial
comp70195_c1	6.91	0.7	3.293927227	9.91E-05	interferon-inducible 58 kDa protein
comp100295_c4	3.84	0.39	3.288644267	3.68E-05	PREDICTED: sacsin
comp54916_c0	3.92	0.41	3.268836246	8.51E-04	Meiosis expressed gene 1 homolog
comp101897_c0	0.78	0.08	3.203998194	1.18E-03	required for meiotic nuclear division protein 1 homolog
comp70117_c0	3.18	0.36	3.15019175	1.39E-03	protein phosphatase 1 regulatory subunit 3D
comp88493_c0	11.5	1.32	3.122446759	2.99E-05	PREDICTED: hypothetical protein LOC794824
comp102789_c0	3.53	0.41	3.110604345	2.85E-05	relA-associated inhibitor
comp101244_c8	3.14	0.36	3.108371574	1.64E-03	neuronal growth regulator 1 precursor
comp102716_c3	5.17	0.61	3.073571692	3.81E-05	PREDICTED: protein FAM151B-like
comp70195_c0	7.93	0.95	3.059043862	5.29E-05	interferon-inducible protein IFI58

Table S1. *Cont.*

Gene ID	RSEM (FW)	RSEM (SM)	Fold Change	*p*-Value	Gene Name
comp59547_c0	9.01	1.09	3.054331734	3.41E-05	IFN-regulatory factory 7
comp102200_c2	9.08	1.1	3.038560751	2.20E-05	PREDICTED: low-density lipoprotein receptor-related protein 1
comp100925_c2	2.67	0.35	2.94240426	1.16E-04	peroxisome proliferator-activated receptor delta b
comp94818_c1	6.64	0.87	2.935576679	7.86E-05	PREDICTED: hypothetical protein LOC100704369
comp102921_c0	3.65	0.49	2.897271329	1.08E-04	UDP glucuronosyltransferase 5 family, polypeptide C1
comp102450_c3	1.31	0.18	2.834712906	5.20E-04	PREDICTED: inositol hexakisphosphate and diphosphoinositol-pentakisphosphate kinase 1-like
comp100801_c4	3.41	0.48	2.828263655	1.43E-03	Zgc:171445 protein
comp99570_c0	1.85	0.27	2.790523239	6.79E-04	3'-5' exoribonuclease 1
comp102423_c4	3.6	0.52	2.786443479	8.77E-04	Si:dkey-95h12.1
comp102293_c8	1.76	0.26	2.782022898	5.03E-04	PREDICTED: histone-lysine N-methyltransferase MLL3
comp97178_c3	5.03	0.74	2.765069829	3.51E-04	PREDICTED: peroxisomal proliferator-activated receptor A-interacting complex 285 kDa protein-like
comp99682_c0	7.94	1.19	2.73240364	4.10E-04	Si:dkey-60b12.4
comp100307_c2	3.11	0.48	2.693334075	1.95E-03	Myxovirus (influenza) resistance A
comp102642_c0	6.26	1	2.651760005	3.85E-04	Fibroblast growth factor receptor substrate 2a
comp102792_c7	0.78	0.12	2.644295994	1.42E-03	formimidoyltransferase-cyclodeaminase
comp101642_c3	6.84	1.11	2.619902023	3.92E-04	folliculin-interacting protein 2
comp100801_c1	3.49	0.57	2.605871234	2.79E-03	alpha-2-macroglobulin-like precursor
comp102641_c3	3.16	0.53	2.579052117	7.44E-04	thyroid transcription factor 1-associated protein 26 homolog
comp102984_c4	2.86	0.48	2.578904186	2.55E-03	tetratricopeptide repeat protein 12
comp69638_c0	4.28	0.72	2.573449756	1.22E-03	Cytokine inducible SH2-containing protein b
comp99986_c0	12.58	2.17	2.533978315	3.62E-04	PREDICTED: hypothetical protein LOC100332522
comp101958_c11	2.74	0.48	2.516001329	7.40E-04	stromal membrane-associated protein 1
comp102423_c1	2.71	0.48	2.492214452	2.66E-03	PREDICTED: hypothetical protein LOC100702146
comp102794_c4	6.18	1.1	2.489761023	9.66E-04	Si:ch211-182e10.4
comp101584_c0	7.63	1.38	2.461854403	6.15E-04	probable ribosome biogenesis protein NEP1
comp95531_c0	1.69	0.31	2.456224697	2.56E-03	enolase superfamily 1
comp100778_c2	7.65	1.41	2.43855899	6.23E-04	PREDICTED: uncharacterized protein C6orf150-like
comp89409_c2	6.49	1.2	2.435946232	2.97E-03	E3 ubiquitin-protein ligase parkin
comp102504_c1	7.76	1.44	2.424652053	5.91E-04	uncharacterized protein LOC692330
comp100578_c1	7.81	1.46	2.421908399	6.26E-04	PREDICTED: probable G-protein coupled receptor-like
comp99726_c0	2.9	0.54	2.418950984	1.19E-03	PREDICTED: protein FAM83C-like
comp100876_c14	10.48	1.97	2.411192795	7.86E-04	PREDICTED: hypothetical protein LOC100707133
comp88384_c0	2.87	0.54	2.400997934	1.92E-03	Replication factor C (activator 1) 3
comp89473_c0	4.71	0.9	2.394367128	1.76E-03	Transmembrane protein 170A
comp102423_c3	4.28	0.81	2.393462901	1.43E-03	PREDICTED: hypothetical protein LOC100703585
comp100309_c0	5.05	0.96	2.39216454	1.70E-03	Rhesus blood group, C glycoprotein a
comp100390_c1	3.22	0.1	2.39216454	1.70E-03	LysM, putative peptidoglycan-binding, domain containing 1
comp97714_c1	4.14	0.79	2.380804678	2.81E-03	Sox9a protein
comp102725_c1	4.37	0.86	2.352041382	1.35E-03	PREDICTED: hypothetical protein LOC100005456
comp102181_c5	5.15	1.02	2.334066618	1.65E-03	Uncharacterized protein
comp102087_c6	2.39	0.5	2.253017148	1.68E-03	suppressor of cytokine signaling 3b

Table S1. *Cont.*

Gene ID	RSEM (FW)	RSEM (SM)	Fold Change	*p*-Value	Gene Name
comp102600_c16	4.19	0.89	2.24035387	2.40E-03	PREDICTED: protein jagged-2-like
comp102818_c3	6.69	1.44	2.211343688	1.49E-03	conserved hypothetical protein
comp101730_c0	1.74	0.38	2.207041996	2.99E-03	nuclear factor, interleukin 3 regulated, member 6
comp102454_c1	2.54	0.55	2.194850964	2.41E-03	ovarian TNF receptor
comp102149_c0	1753.31	384.13	2.190412697	1.32E-03	PREDICTED: hypothetical protein LOC556125
comp69987_c0	6.11	1.35	2.182719163	2.56E-03	tether containing UBX domain for GLUT4
comp103013_c11	10.8	2.42	2.15545804	1.77E-03	Myeloid cell leukemia sequence 1b
comp102484_c6	5.73	1.29	2.153057175	2.37E-03	cholesteryl ester transfer protein precursor
comp101798_c0	22.53	5.14	2.133004444	1.85E-03	hydroxymethylglutaryl-CoA synthase, cytoplasmic
comp101939_c4	169.91	40.48	2.069303681	2.32E-03	Ceruloplasmin
comp69666_c0	308.35	75.27	2.034382341	2.72E-03	Sepp1a protein
comp103171_c0	195.29	796	−2.027156846	2.81E-03	novel elastase protein (zgc:637440)
comp68911_c0	765.1	3125.68	−2.030452641	2.76E-03	Try protein
comp103180_c0	163.6	671.79	−2.037874358	2.69E-03	PREDICTED: syncollin-like
comp102210_c0	500.1	2087.51	−2.061493791	2.40E-03	pancreatic elastase precursor
comp62899_c0	174.73	753.77	−2.109004544	1.94E-03	six-cysteine containing astacin protease 3 precursor
comp61984_c0	1.63	7.04	−2.113256505	2.80E-03	Uncharacterized protein
comp71713_c0	12.89	56.21	−2.124195595	1.87E-03	glucokinase
comp115558_c0	2.87	12.53	−2.126989962	3.07E-03	fep15 selenoprotein precursor
comp102032_c6	1.09	4.79	−2.136963066	2.19E-03	PREDICTED: c-Jun-amino-terminal kinase-interacting protein 4-like
comp102458_c14	6.85	30.38	−2.149483538	1.76E-03	steroid hormone receptor ERR1
comp102451_c3	6.6	29.44	−2.156706265	1.64E-03	putative hexokinase HKDC1
comp62088_c0	10.95	48.83	−2.15730424	1.90E-03	PREDICTED: elastase inhibitor-like
comp100340_c0	4.94	22.06	−2.158703255	1.77E-03	squalene synthase
comp103030_c2	0.79	3.55	−2.165308674	2.09E-03	HEAT repeat-containing protein 1
comp102715_c4	1.39	6.23	−2.169065254	1.98E-03	PREDICTED: im:7147678
comp64707_c0	4.5	20.54	−2.189875445	1.56E-03	probable phosphatase phospho1
comp102051_c0	8.66	40.14	−2.212896955	1.30E-03	glutathione S-transferase theta-like
comp100511_c0	18.78	87.63	−2.222350104	1.21E-03	poly(U)-specific endoribonuclease-A precursor
comp102431_c0	5.5	26.35	−2.259400273	1.11E-03	PREDICTED: hypothetical protein LOC557984
comp103680_c0	39.68	192.49	−2.278226163	9.08E-04	Si:dkey-14d8.7 protein
comp102458_c8	3.97	19.3	−2.280260802	1.36E-03	estrogen-related receptor alpha
comp101902_c0	6.28	30.99	−2.30267668	9.04E-04	DNase I
comp102936_c9	2.41	11.91	−2.304175853	8.63E-04	malic enzyme 1, NADP(+)-dependent, cytosolic
comp102546_c1	0.43	2.14	−2.311099788	1.55E-03	Interleukin enhancer binding factor 3b
comp71193_c0	0.81	4.06	−2.322499089	1.35E-03	protein phosphatase 1 regulatory subunit 3B
comp100850_c0	351.26	1763.83	−2.328081045	6.85E-04	chymotrypsin B1 precursor
comp103032_c0	0.97	4.89	−2.332728222	2.51E-03	PREDICTED: EMILIN-3
comp100683_c0	0.26	1.33	−2.346273655	2.47E-03	cysteinyl-tRNA synthetase, cytoplasmic
comp102908_c10	1.01	5.19	−2.361560904	3.02E-03	Si:dkeyp-2c8.3

Table S1. *Cont.*

Gene ID	RSEM (FW)	RSEM (SM)	Fold Change	*p*-Value	Gene Name
comp102905_c13	2.48	12.78	−2.36442847	7.85E-04	mitochondrial 39S ribosomal protein l16
comp102530_c7	4.94	25.53	−2.371096909	6.38E-04	heparin cofactor 2 precursor
comp97270_c0	0.61	3.16	−2.381189711	1.30E-03	PREDICTED: hypothetical protein LOC100001663
comp89363_c0	0.34	1.8	−2.415246436	2.94E-03	dehydrodolichyl diphosphate synthase
comp102127_c8	0.75	4.11	−2.446715728	1.43E-03	novel protein similar to vertebrate COMM domain containing 9 (COMMD9)
comp102950_c1	0.47	2.59	−2.449201757	7.86E-04	Chloride channel 2
comp103123_c0	218.85	1200.42	−2.455500558	3.65E-04	Amylase, alpha 2A; pancreatic
comp101235_c1	62.8	345.94	−2.461718237	3.53E-04	PREDICTED: fatty acid synthase-like
comp103032_c10	0.78	4.31	−2.475517093	1.85E-03	Elastin microfibril interfacer 3a
comp97726_c1	1.23	6.88	−2.486051256	1.20E-03	Proteasome (prosome, macropain) subunit, beta type, 10
comp89158_c1	0.95	5.38	−2.502653223	6.86E-04	PREDICTED: calmegin-like
comp102176_c10	3.82	0	−2.517647961	7.75E-04	PREDICTED: hypothetical protein LOC100535924
comp101289_c0	0.28	1.59	−2.521283793	1.31E-03	interleukin 7 receptor precursor
comp66504_c0	0.89	5.13	−2.523411783	1.88E-03	PREDICTED: microfibril-associated glycoprotein 4-like
comp103534_c0	49.79	289.66	−2.54028155	2.44E-04	chymotrypsin-like precursor
comp88432_c0	1.54	9.21	−2.576653805	4.17E-04	Fgf19 protein
comp102964_c0	2.19	13.19	−2.587761059	2.38E-04	uncharacterized protein LOC553197
comp102615_c10	61.75	377.48	−2.611993436	1.66E-04	Cpb1 protein, partial
comp102803_c0	62.54	384.35	−2.61967196	1.59E-04	Cel.2 protein
comp94017_c3	0.56	3.47	−2.622947457	2.47E-03	uncharacterized protein LOC393586
comp102620_c7	0.27	1.65	−2.631568212	1.83E-03	protein SSXT
comp101599_c0	0.24	1.49	−2.657404962	6.98E-04	PREDICTED: dihydroxyacetone phosphate acyltransferase-like
comp93327_c1	26.79	171.69	−2.680027069	1.21E-04	Hsp90a.2 protein
comp100272_c0	0.44	2.84	−2.691603817	5.21E-04	PREDICTED: tyrosine-protein kinase BAZ1B
comp100350_c0	0.63	4.12	−2.719089046	2.83E-04	protein phosphatase 1, regulatory (inhibitor) subunit 14Ab
comp93327_c0	34.06	224.4	−2.7198656	9.86E-05	heat shock protein 90, alpha (cytosolic), class A member 1, tandem duplicate 2
comp93706_c0	0.35	2.35	−2.752897531	8.63E-04	Transcription elongation factor, mitochondrial
comp93536_c0	0.58	3.95	−2.756698846	1.84E-03	PREDICTED: hypothetical protein LOC100150934
comp101238_c1	0.39	2.63	−2.764894846	6.72E-04	suppressor of G2 allele of SKP1 homolog
comp101398_c0	0.44	3.15	−2.827367831	2.22E-04	PREDICTED: nucleotide exchange factor SIL1-like
comp102173_c5	2.47	17.76	−2.848157098	5.31E-05	PREDICTED: nodal modulator 1
comp100723_c0	0.04	0.31	−2.851233082	2.76E-03	G/T mismatch-specific thymine DNA glycosylase
comp93327_c2	5.29	39.98	−2.919385021	7.45E-05	PREDICTED: heat shock protein HSP 90-alpha-like
comp101217_c0	0.68	5.19	−2.923949713	1.37E-04	PREDICTED: uncharacterized protein LOC575262
comp100319_c0	0.28	2.14	−2.925292273	2.25E-04	PREDICTED: hypothetical protein LOC558212
comp101669_c0	0.12	0.93	−2.932418175	1.31E-03	PREDICTED: e3 ubiquitin/ISG15 ligase TRIM25
comp69063_c0	0.46	3.55	−2.942565391	1.82E-03	uncharacterized protein LOC793030 precursor
comp101051_c0	0.69	5.42	−2.973769627	1.32E-04	ubiquitin C-terminal hydrolase L1
comp100438_c0	3.38	26.77	−2.985973242	2.49E-05	Ddx5 protein
comp102982_c3	0.12	1.03	−3.050368681	3.45E-04	replication protein A 70 kDa DNA-binding subunit

Table S1. *Cont.*

Gene ID	RSEM (FW)	RSEM (SM)	Fold Change	*p*-Value	Gene Name
comp69999_c1	0.38	3.15	−3.054379394	4.97E-04	polyadenylate-binding protein 4
comp102748_c0	1.97	17.5	−3.15481999	1.19E-05	DnaJ (Hsp40) homolog, subfamily B, member 1
comp102507_c0	0.46	4.1	−3.162359597	1.91E-05	ER degradation-enhancing alpha-mannosidase-like 1
comp91343_c0	0.29	2.66	−3.201483688	2.55E-03	GTP binding protein 1, like
comp102526_c5	0.68	6.3	−3.211687468	2.25E-05	guanylate binding protein 4
comp89910_c0	1.34	13.12	−3.286315718	1.05E-05	mid1-interacting protein 1-like
comp103002_c6	0.42	4.11	−3.301019362	2.31E-04	PREDICTED: hypothetical protein LOC100498431
comp99919_c0	0.19	1.89	−3.301019362	1.72E-03	Zinc finger protein 503
comp102119_c5	0.38	3.77	−3.303455661	1.49E-05	PREDICTED: plexin-C1
comp102659_c8	0.66	6.67	−3.347015692	1.18E-05	PREDICTED: receptor-type tyrosine-protein phosphatase zeta
comp102832_c7	25.17	257.08	−3.352569943	2.82E-06	PREDICTED: maltase-glucoamylase, intestinal
comp100688_c0	0.36	3.83	−3.394128766	1.18E-03	gamma-glutamyl cyclotransferase a
comp102786_c1	0.91	9.64	−3.411500326	3.38E-06	PREDICTED: lon protease homolog, mitochondrial
comp102401_c0	0.3	3.35	−3.462783801	6.81E-06	PREDICTED: baculoviral IAP repeat-containing protein 6
comp102538_c1	0.19	2.17	−3.502653223	2.81E-03	PREDICTED: phosphofurin acidic cluster sorting protein 2-like
comp93835_c1	0.23	2.56	−3.502653223	8.70E-05	conserved hypothetical protein
comp97468_c1	0.28	3.24	−3.533680119	2.56E-04	superoxide dismutase-like
comp101358_c1	0.23	2.78	−3.622947457	1.77E-03	tripartite motif containing 35
comp99683_c0	0.2	2.5	−3.644269245	2.28E-05	cleavage and polyadenylation specificity factor subunit 6
comp102656_c0	0.22	2.82	−3.648951473	8.99E-06	PREDICTED: hypothetical protein LOC100698500
comp60482_c0	0.23	3.17	−3.786446189	9.21E-04	IGF
comp89158_c0	0.4	5.6	−3.792817277	2.45E-05	Si:ch211-274f20.2
comp102529_c8	1.33	18.57	−3.80445941	4.26E-07	trans-2,3-enoyl-CoA reductase
comp115872_c0	0.99	14.84	−3.907198349	3.32E-06	metallothionein B-like
comp101706_c1	0.28	4.36	−3.964637478	4.73E-07	protein NipSnap homolog 2
comp102184_c1	3.91	61.24	−3.969076109	8.08E-08	PREDICTED: maltase-glucoamylase, intestinal-like
comp102942_c7	0.15	2.59	−4.112257718	2.61E-05	PREDICTED: hypothetical protein LOC100692678
comp89027_c0	0.39	6.92	−4.151318735	4.48E-08	PREDICTED: uncharacterized protein LOC763830, partial
comp98089_c0	6.06	112.25	−4.21203887	2.32E-08	apolipoprotein D-like precursor
comp92587_c0	0.1	1.82	−4.22701878	2.29E-03	Solute carrier family 28
comp98365_c5	0.11	2.25	−4.301019362	1.74E-03	growth hormone receptor
comp102403_c1	0.06	1.3	−4.35459414	1.34E-05	ELMO/CED-12 domain containing 2 precursor
comp70923_c2	0.14	2.83	−4.37140869	1.33E-03	PREDICTED: erythroid differentiation-related factor 1-like
comp102628_c2	2.38	49.59	−4.382899911	6.70E-09	heat shock 70 kDa protein
comp101759_c0	0.04	0.82	−4.430485567	1.13E-04	PREDICTED: probable ergosterol biosynthetic protein 28-like
comp101274_c13	0.12	2.67	−4.506120051	2.20E-05	PREDICTED: hypothetical protein LOC560266
comp96421_c3	0.19	4.48	−4.546775776	1.85E-07	uncharacterized protein LOC567731 precursor
comp101181_c0	0.23	6.66	−4.847549117	1.40E-09	PREDICTED: vitellogenin

Table S1. *Cont.*

Gene ID	RSEM (FW)	RSEM (SM)	Fold Change	*p*-Value	Gene Name
comp83201_c0	0.34	10.07	−4.885981863	1.04E-08	Retinoic acid receptor responder protein 3
comp94015_c0	0.12	4.28	−5.188544633	9.85E-07	PREDICTED: UHRF1-binding protein 1-like
comp93355_c3	0.07	2.59	−5.264493486	2.69E-05	unnamed protein product
comp101279_c2	0.22	8.49	−5.267673548	2.45E-10	rho-related gtp-binding protein rhoc
comp100318_c1	0.05	2.26	−5.438522886	1.14E-05	myosin heavy chain
comp101504_c1	0.03	1.67	−5.740474884	5.79E-07	SRF6-like
comp69517_c0	0.59	31.7	−5.752345744	2.92E-12	aspartyl-tRNA synthetase, cytoplasmic
comp102814_c2	0.03	1.62	−5.759816224	4.03E-07	putative L-aspartate dehydrogenase
comp102544_c0	0.01	1.3	−6.504942325	4.54E-07	PREDICTED: phosphatidylinositol 3-kinase regulatory subunit alpha isoform 1
comp101847_c9	0.19	21.71	−6.861734316	3.85E-09	Cytochrome P450, family 2, subfamily K, polypeptide 6
comp102894_c4	0.08	10.92	−7.025669634	9.43E-14	PREDICTED: hypothetical protein LOC100710859
comp99707_c1	0.15	20.31	−7.087615724	9.61E-10	hypothetical protein SERLA73DRAFT_67379
comp70577_c0	0.1	15.4	−7.212710944	4.29E-12	PREDICTED: hypothetical protein LOC100333953
comp102199_c0	0.36	56.41	−7.292632585	3.12E-16	carboxypeptidase A precursor
comp101826_c2	0.02	3.35	−7.696151222	9.81E-13	serine/threonine-protein kinase Sgk1
comp102799_c4	0	0.96	−11.03233839	1.65E-04	zgc:123060（ZFIN）
comp102863_c0	0	2.03	−13.08221512	1.12E-11	BSD domain containing 1
comp102301_c13	0	2.19	−29.10443731	2.86E-03	probable E3 ubiquitin-protein ligase MYCBP2
comp56672_c0	0	1.93	−29.10443731	2.86E-03	dnaJ homolog subfamily C member 17
comp68552_c0	0	1.78	−29.10443731	2.86E-03	uncharacterized protein LOC563088
comp92255_c2	0	1.86	−29.10443731	2.86E-03	novel protein similar to vertebrate IMP (inosine monophosphate) dehydrogenase 1 (IMPDH1)
comp96783_c2	0	1.75	−29.10443731	2.86E-03	myosin light chain kinase 3
comp99266_c0	0	1.75	−29.10443731	2.86E-03	PREDICTED: early endosome antigen 1
comp101708_c0	0	0.41	−29.11659337	2.00E-03	solute carrier family 25 member 47-B
comp102975_c0	0	0.3	−29.21032165	2.00E-03	cysteine and histidine-rich domain-containing protein 1
comp90049_c0	0	1.72	−29.21135252	2.00E-03	si:dkeyp-52c3.2
comp95917_c1	0	1.71	−29.21135252	2.00E-03	PREDICTED: trinucleotide repeat-containing gene 6B protein
comp96313_c2	0	2.12	−29.21135252	2.00E-03	putative transcription factor COE1
comp101498_c2	0	0.74	−29.32238383	1.01E-03	uncharacterized protein LOC100037365
comp80071_c2	0	2.94	−29.40399759	1.01E-03	gamma-glutamylcyclotransferase precursor
comp102784_c0	0	0.99	−29.44751709	7.27E-04	Transposable element Tcb1 transposase
comp98627_c2	0	1.31	−29.46922522	7.27E-04	Zgc:110391 protein, partial
comp71302_c0	0	2.37	−29.49146044	7.27E-04	zgc:101901
comp98392_c1	0	1.83	−29.49146044	7.27E-04	Zinc finger, BED-type containing 1
comp97430_c0	0	1.09	−29.50497524	5.30E-04	Salt-inducible kinase 2a
comp100878_c0	0	0.73	−29.54967505	5.30E-04	PREDICTED: hypothetical protein LOC100693263
comp96436_c0	0	2.39	−29.5739226	5.30E-04	ORF2-encoded protein
comp98885_c2	0	1.62	−29.5811181	5.30E-04	PREDICTED: sorbin and SH3 domain-containing protein 2
comp102854_c2	0	0.08	−29.63050612	3.91E-04	E3 ubiquitin-protein ligase HECTD1
comp101583_c0	0	0.89	−29.63972465	3.91E-04	PREDICTED: hypothetical protein LOC100697633

Table S1. *Cont.*

Gene ID	RSEM (FW)	RSEM (SM)	Fold Change	*p*-Value	Gene Name
comp102412_c2	0	3.61	−29.65192511	3.91E-04	PREDICTED: microfibril-associated glycoprotein 4
comp56282_c0	0	2.26	−29.65192511	3.91E-04	invariant chain like protein 1
comp93358_c0	0	1.8	−29.65192511	3.91E-04	probable alpha-ketoglutarate-dependent dioxygenase ABH6
comp102966_c5	0	0.25	−29.67676733	2.91E-04	RAC-alpha serine/threonine-protein kinase
comp102570_c4	0	0.39	−29.7601414	2.18E-04	isobutyryl-CoA dehydrogenase, mitochondrial
comp102114_c11	0	0.48	−29.80521846	1.65E-04	PREDICTED: cell death activator CIDE-A-like
comp98762_c1	0	1.37	−29.83026235	1.65E-04	Wilms tumor suppressor protein 1b
comp89799_c0	0	1.05	−29.93818373	9.64E-05	transcription elongation factor B polypeptide 1
comp102835_c2	0	0.87	−29.95551361	9.64E-05	PREDICTED: band 4.1-like protein 2-like, partial
comp102865_c13	0	0.88	−29.97446053	9.64E-05	PREDICTED: centromere protein V
comp91465_c0	0	3.39	−29.9889601	9.64E-05	zona pellucida glycoprotein 2.1
comp97205_c0	0	2.55	−29.9889601	9.64E-05	PREDICTED: transposon TX1 uncharacterized 149 kDa protein-like
comp90899_c1	0	2.78	−30.04785378	7.45E-05	PREDICTED: hypothetical protein LOC568313
comp97112_c0	0	0.46	−30.06335135	5.79E-05	Aldehyde dehydrogenase 9 family, member A1b
comp102593_c7	0	3.9	−30.10443731	5.79E-05	hypothetical protein MYCTH_2071514
comp98392_c0	0	2.37	−30.1588851	4.53E-05	PREDICTED: hypothetical protein LOC100700880
comp102406_c2	0	0.97	−30.28076009	2.25E-05	MAP7 domain-containing protein 1
comp102473_c3	0	1.13	−30.29005993	2.25E-05	PREDICTED: wolframin
comp102583_c12	0	1.74	−30.30220595	2.25E-05	PREDICTED: leukocyte elastase inhibitor isoform 1
comp99076_c1	0	1.86	−30.31088819	2.25E-05	uncharacterized protein LOC791929
comp99905_c1	0	2.96	−30.31088819	2.25E-05	PREDICTED: suppressor of tumorigenicity 14 protein homolog
comp93835_c2	0	2.98	−30.3581939	1.80E-05	PREDICTED: uncharacterized protein LOC100890688
comp89898_c0	0	1.09	−30.3931366	1.45E-05	PREDICTED: hypothetical protein LOC100534830
comp103011_c7	0	2.82	−30.40399759	1.45E-05	PREDICTED: hypothetical protein LOC553357
comp95528_c0	0	2.95	−30.40399759	1.45E-05	MHC class II invariant chain Ii
comp101618_c7	0	0.41	−30.43035504	1.17E-05	oxysterol binding protein-like 3a
comp92178_c1	0	1.28	−30.50623579	7.72E-06	integrator complex subunit 10
comp102522_c0	0	0.7	−30.56023239	6.32E-06	uncharacterized protein LOC100141491 precursor
comp102274_c0	0	0.44	−30.56346532	6.32E-06	PREDICTED: protein FAM13A isoform 1
comp102156_c16	0	1.63	−30.58271224	6.32E-06	PREDICTED: oxysterol-binding protein-related protein 10-like, partial
comp102014_c2	0	0.3	−30.5957971	5.19E-06	protein transport protein Sec24C
comp102006_c3	0	3.44	−30.59658632	5.19E-06	PREDICTED: hypothetical protein LOC100332181
comp99707_c0	0	7.65	−30.79631502	2.05E-06	Zgc:158463
comp101827_c2	0	0.59	−30.80794696	1.72E-06	Eukaryotic translation initiation factor 5
comp100128_c4	0	0.74	−30.84627763	1.45E-06	fat storage-inducing transmembrane protein 2
comp97699_c1	0	3.06	−30.86342921	1.45E-06	Zgc:174680
comp102379_c11	0	0.93	−30.88264589	1.22E-06	Tryptophan 2,3-dioxygenase a
comp99142_c1	0	0.88	−30.88974642	1.22E-06	NEDD8
comp60157_c0	0	3.5	−30.89585069	1.22E-06	uncharacterized protein LOC100327252 precursor

Table S1. *Cont.*

Gene ID	RSEM (FW)	RSEM (SM)	Fold Change	*p*-Value	Gene Name
comp102058_c22	0	1.12	−30.96562922	8.76E-07	PREDICTED: ribosomal protein S6 kinase beta-1-like
comp102551_c5	0	0.68	−30.98233254	7.45E-07	predicted protein
comp96828_c2	0	2.49	−31.04785378	5.43E-07	PREDICTED: protein transport protein Sec31A
comp99967_c1	0	1.43	−31.13382224	3.45E-07	PREDICTED: pleckstrin homology domain-containing family G member 5
comp102593_c18	0	8.46	−31.1588851	2.97E-07	hypothetical protein PABG_06807
comp102492_c1	0	1.21	−31.17983829	2.57E-07	PREDICTED: collagen alpha-1(XII) chain-like
comp93355_c6	0	3.41	−31.18535731	2.57E-07	PREDICTED: LOW QUALITY PROTEIN: wu:fl04e06
comp92949_c0	0	2.55	−31.23688761	1.94E-07	PREDICTED: hypothetical protein LOC100537292
comp101409_c1	0	2.02	−31.57872358	2.87E-08	DNA damage-inducible transcript 4 protein
comp102519_c6	0	1.73	−31.63243151	2.04E-08	PREDICTED: hypothetical protein LOC100538074
comp101021_c0	0	5.2	−31.75396434	9.62E-09	PREDICTED: urokinase plasminogen activator surface receptor-like
comp88529_c1	0	2.22	−31.75714442	9.62E-09	gap junction beta-6 protein
comp102665_c1	0	0.64	−31.81525434	7.08E-09	PREDICTED: protein cordon-bleu
comp102794_c5	0	1.06	−31.8563624	5.80E-09	abhydrolase domain-containing protein 4
comp89370_c0	0	4.99	−31.86277329	5.26E-09	UPF0542 protein C5orf43 homolog
comp102887_c12	0	1.05	−31.91669856	3.94E-09	PREDICTED: heterogeneous nuclear ribonucleoprotein U-like protein 1-like
comp102620_c6	0	1.34	−31.93272518	3.58E-09	nucleolysin TIAR
comp101302_c0	0	13.32	−32.01870744	2.07E-09	cytochrome oxidase subunit I (mitochondrion)
comp102816_c11	0	1.63	−32.06363678	1.59E-09	Activated leukocyte cell adhesion molecule
comp100712_c0	0	2.99	−32.11659337	1.13E-09	protein IMPACT
comp102652_c1	0	1.45	−32.1837826	7.51E-10	mucolipin-2
comp100875_c0	0	1.45	−32.30908369	3.47E-10	CD2-associated protein
comp102883_c1	0	1.23	−32.33556247	2.99E-10	complement receptor-like protein precursor
comp102724_c0	0	1.13	−32.3398109	2.99E-10	PREDICTED: transcriptional repressor NF-X1-like
comp102106_c0	0	2.77	−32.34381185	2.99E-10	Rnu3ip2 protein
comp102866_c2	0	2.14	−32.35259844	2.78E-10	PREDICTED: ankyrin repeat and SAM domain-containing protein 1A-like
comp102450_c7	0	1.79	−32.3909773	2.24E-10	45 kDa calcium-binding protein precursor
comp102566_c14	0	3.23	−32.4246998	1.81E-10	uncharacterized protein LOC559970
comp101818_c1	0	1.45	−32.46198932	1.38E-10	uncharacterized protein LOC564531
comp102593_c22	0	14.52	−32.47008678	1.29E-10	hypothetical protein AN5245.2
comp96751_c0	0	1.26	−32.49833924	1.13E-10	PREDICTED: pantothenate kinase 4-like
comp102462_c1	0	1.41	−32.56548223	7.15E-11	PREDICTED: protein sidekick-1
comp126569_c0	0	6.74	−32.62316611	5.23E-11	ATP synthase F0 subunit 6 (mitochondrion)
comp102651_c0	0	3.18	−32.66336425	3.86E-11	PREDICTED: si:ch211-197g15.7
comp101847_c3	0	20.27	−32.82185011	1.47E-11	uncharacterized protein LOC100170784
comp98419_c0	0	6.87	−32.82185011	1.47E-11	cytochrome b
comp99666_c0	0	4.89	−32.89504897	9.12E-12	COP9 signalosome complex subunit 4
comp89974_c0	0	7.01	−32.9984715	4.76E-12	ATPase, H+ transporting, V1 subunit F

Table S1. *Cont.*

Gene ID	RSEM (FW)	RSEM (SM)	Fold Change	*p*-Value	Gene Name
comp101806_c1	0	4.16	−33.00532269	4.54E-12	PREDICTED: FAST kinase domain-containing protein 5-like
comp125637_c0	0	12.37	−33.06220908	3.11E-12	cytochrome oxidase subunit II
comp100614_c4	0	3.71	−33.07217346	2.97E-12	Protein tyrosine phosphatase, receptor type, C
comp102940_c2	0	1.83	−33.2955383	6.90E-13	UHRF1-binding protein 1-like
comp102395_c1	0	5.76	−33.31130891	6.12E-13	PREDICTED: zinc-binding protein A33-like
comp102593_c16	0	15.14	−33.44839171	2.48E-13	transcript antisense to ribosomal RNA protein
comp102514_c1	0	5.67	−33.50739032	1.68E-13	Uncharacterized protein
comp99321_c0	0	4.59	−33.59238863	9.79E-14	V-fos FBJ murine osteosarcoma viral oncogene homolog
comp99121_c0	0	15.98	−33.7482935	3.50E-14	Cytochrome c oxidase III, mitochondria
comp102592_c0	0	2.32	−33.77202381	2.93E-14	G protein-coupled receptor 56 precursor
comp102167_c10	0	4.32	−34.00881024	6.16E-15	novel protein similar to vertebrate gliacolin (C1Q)
comp101302_c1	0	20.44	−34.05145602	4.59E-15	cytochrome oxidase subunit I (mitochondrion)
comp102901_c1	0	3.82	−34.13215621	2.67E-15	PREDICTED: Hypothetical protein LOC100004401
comp99594_c0	0	4.24	−34.13548634	2.61E-15	phosphatidylinositide phosphatase SAC1-B
comp101702_c0	0	20.99	−34.19516048	1.78E-15	Ependymin
comp97193_c3	0	6.41	−34.43074233	3.66E-16	acyl-CoA-binding domain-containing protein 5A
comp102884_c12	0	7.05	−34.52532389	1.94E-16	E3 ubiquitin-protein ligase LRSAM1
comp102221_c5	0	5.19	−34.5539225	1.57E-16	G protein-coupled receptor 161
comp102354_c4	0	14.11	−34.77152155	3.65E-17	DDB1- and CUL4-associated factor 13
comp70508_c0	0	9.82	−34.84278945	2.23E-17	β-centractin
comp99354_c0	0	14.7	−35.45492096	3.46E-19	casein kinase 2 α 1
comp102644_c2	0	29.27	−36.0859036	4.61E-21	Golgi transport 1 homolog B

Table S2. Primers used for qRT-PCR validation.

Gene ID	Gene Name	Primers Sequences	
		**Sense 5′–3′**	**Anti-Sense 5′–3′**
comp103013_c11	MCL1b	TCACACGGTTCTACTGTGTG	CATCATGTTCCGATCCTTCC
comp102279_c14	PPM1K	TCAGCTATCGCAGATGACCG	GTGAAGATGCCTGGCTAACG
comp102791_c0	C4	AGGCACTGAACAGCAGATAG	TGCTTCCTCTAGATTGCCAC
comp102564_c2	STAT6	TTCAGAACTGCCGACAAACG	GAAGAAGCTGCAAACGCTTC
comp93418_c0	YAF2	GGAAGTGACAGTGGGAGATC	GTGAGACTCTCCGTTGAGTG
comp101330_c3	SDR16C5	TGAAGCGTGAGGTTGGTGAC	GATGAGTCCAGCAGAACTCG
comp102898_c4	MCM5	GGTCGTGATGGTCTTTGATG	GAGCAGCAGACCATCTCTAT
comp89409_c2	Park2	AGAACGGTGGCTGTATGCAC	CCCATTGGTTCCATTCCCTG
comp102628_c2	Hap70	AAGAGAACCCTCTCCTCCAG	GCTTTCTCCACAGGCTCAAG
comp102986_c4	Cyp2p9	GGTGAAGAGAAAGTGAAGCGC	GATCACTTCCTGGATGCTGAG
comp102087_c6	Socs3b	ATGAGGCAGCTGGTCCCGTT	GGAGACCTCTTCGAAGGTCC
comp102484_c6	CETP	GACATCTCTGTGACCTCTTC	CATTAAGGAGTCAAGGAGCG
comp60482_c0	IGF	GTCAGCCACCTCAGTTTCCT	TCCAGTGTGAACGCTAACGC
comp102814_c2	ASPDH	AGTGGAGGTGTGTCATCCAC	CCACCCCATAATGCACCACT
comp101847_c9	Cyp2k6	TTGACACAACTCAGCCTGTG	TAAGCTCGTCTGACCATGTG
comp102544_c0	PIK3	AACCAGGGATGTGTGCTGGT	TCAGGGACAGCAGCAAACCT
comp99124_c0	Adm2a	AACATGAGGCTCTCCCTGTG	GGCGCTAATCTGCTCAAAGG
comp102572_c6	C3c	AATCCTTGTACACCACCCGC	CGAGCAGCACATGGCTGATT
comp98419_c0	Cytb	GGGAGAACAGTGCTAGTAGT	AACCATCATCCACCTGCTGT
comp102860_c0	IL13RA1	GGCTTGTGAGGCACATTGTT	ATCTCTTCCACCACCAGAGT
Reference	β-actin	TGCAAAGCCGGATTCGCTGG	AGTTGGTGACAATACCGTGC
